# c-Abl/TFEB Pathway Activation as a Common Pathogenic Mechanism in Lysosomal Storage Diseases: Therapeutic Potential of c-Abl Inhibitors

**DOI:** 10.3390/antiox14050611

**Published:** 2025-05-20

**Authors:** Miguel V. Guerra, Juan Castro, Antonio Moreno, Elisa Balboa, Juan J. Marugan, Alejandra R. Alvarez, Silvana Zanlungo

**Affiliations:** 1 Department of Gastroenterology, Faculty of Medicine, Pontificia Universidad Católica de Chile, Santiago 8331010, Chile; miguel.guerra@uc.cl (M.V.G.); jcastros@uc.cl (J.C.); morenoantonio60@uc.cl (A.M.); 2 Center for Biomedical Research, Universidad Finis Terrae, Santiago 7501015, Chile; ebalboa@uft.cl; 3 Early Translation Branch, National Center for Advancing Translational Sciences (NCATS), National Institute of Health (NIH), 9800 Medical Center Drive, Rockville, MD 20850, USA; maruganj@mail.nih.gov; 4 Cell Signaling Laboratory, Biological Sciences Faculty, Pontificia Universidad Católica de Chile, Santiago 8331150, Chile; 5 Millennium Institute on Immunology and Immunotherapy, Biological Sciences Faculty, Pontificia Universidad Católica de Chile, Santiago 8331150, Chile

**Keywords:** lysosomal storage diseases, Niemann-Pick, Gaucher, c-Abl, transcription factor EB, α-Tocopherol, Neurotinib, Imatinib

## Abstract

Lysosomal storage diseases (LSDs) are characterized by the accumulation of undegraded substrates within lysosomes, often associated with oxidative stress and impaired lysosomal function. In this study, we investigate the role of the c-Abl/TFEB pathway in different LSDs: Gaucher, Niemann-Pick type A (NPA), and Niemann-Pick type C (NPC). Our findings identify c-Abl activation (p-c-Abl) as a common pathogenic mechanism in these disorders. We demonstrate that c-Abl phosphorylates TFEB at Tyr173, leading to its cytoplasmic retention. Using pharmacological models of Gaucher, NPA and NPC in SH-SY5Y neuronal cells and HeLa cells, we assess the effects of the c-Abl inhibitors Imatinib and Neurotinib, as well as the antioxidant α-Tocopherol (α-TOH), on TFEB nuclear translocation and p-c-Abl protein levels. Additionally, we explore the effects of c-Abl inhibitors in cholesterol accumulation in LSDs neuronal models. Our results show that treatment with c-Abl inhibitors or α-TOH promotes TFEB nuclear translocation, enhances lysosomal clearance, and reduces cholesterol accumulation in all three LSD models. These findings highlight the c-Abl/TFEB pathway as a potential therapeutic target for LSDs and potentially other neurodegenerative disorders associated with lysosomal dysfunction.

## 1. Introduction

Lysosomes are key organelles involved in fundamental cellular processes, including the degradation of a variety of substrates, plasma membrane repair, and autophagy [1,2]. Therefore, lysosomes are highly connected with the cellular environment and can sense, adapt, and respond to changes in substrate metabolism maintaining cellular homeostasis [1,2]. Lysosomal dysfunction and impaired lysosomal clearance are emerging as common pathogenic mechanism in neurodegenerative diseases such Parkinson’s disease (PD) and Alzheimer’s disease (AD) [3,4]. Indeed, mutations in the *GBA1* gene, encoding lysosomal β-glucocerebrosidase (GCase), have been identified as major risk factor for PD, emphasizing the critical role of lysosomal integrity in neural survival [5].

In this context, the transcription factor EB (TFEB) plays a crucial role. TFEB belongs to the microphthalmia family of basic helix-loop-helix leucine zipper (bHLH-Zip) transcription factors [6,7] and is capable of activating more than 50 genes that are part of the CLEAR (Coordinated Lysosomal Expression and Regulation) network, involved in lysosomal biogenesis, exocytosis, and autophagy [8]. TFEB activation increases lysosome number and degradative capacity, while promoting autophagic flux and lysosomal exocytosis [9,10,11], stimulating the clearance of substrates stored in cells. TFEB is also activated by different cellular stress conditions, including oxidative stress [12,13,14,15], which in turn induces the expression of antioxidant TFEB target genes, facilitating the elimination of waste material and favoring catabolic processes that restore energy homeostasis [16].

The activity of TFEB as a transcription factor depends on its subcellular localization controlled by its post-translational modifications. Phosphorylation is the main TFEB modification that modulates its translocalization between the cytoplasm and nucleus [2,7]. The mammalian target of rapamycin complex 1 (mTORC1) is a serine/threonine-specific protein kinase (Ser/Thr-Kinase) that regulates TFEB in response to the cellular nutrient status [2,17]. At the molecular level, under conditions of abundant nutrients and optimal state of the lysosome, the mTORC1 kinase complex and TFEB are recruited to the lysosomal membrane, resulting in TFEB phosphorylation at Ser 122, 138, 142, and 211, as well as its cytoplasmic retention [18,19,20]. Other Ser/Thr-Kinases, such as glycogen synthase kinase-3 beta (GSK3β), the extracellular signal-regulated kinase (ERK1/2), and protein kinase B (AKT), have been described being able of phosphorylating TFEB as result of different stimulus, resulting in its inactivation and cytoplasmic retention [21,22,23]. Additionally, TFEB dephosphorylation plays a significant role in modulating the dynamic of nuclear import and export. In situations of lack of nutrients or increased reactive oxygen species (ROS), Ca^2+^ is released from the lysosome, activating the phosphatase Calcineurin, which dephosphorylates TFEB at Ser211, increasing its nuclear localization and activity [15,24].

We have shown that the activity of c-Abl tyrosine (Tyr) kinase regulates TFEB nuclear localization and function in a mTORC1-independent manner [25]. Our previous results suggested that c-Abl mediated TFEB phosphorylation on Tyr173 is relevant for its retention in the cytoplasm [25]. Our results support that c-Abl/TFEB signaling is a new axis that regulates lysosomal biogenesis and function. c-Abl is activated under pathophysiological conditions, including Lysosomal Storage Diseases (LSDs), such as Niemann-Pick A (NPA), Niemann-Pick type C (NPC), and Gaucher [26,27,28,29]. Interestingly, c-Abl inhibition in NPC models increases TFEB nuclear levels and promotes cellular clearance, reducing cholesterol accumulation in lysosomes, a characteristic feature of NPC cells. This TFEB-dependent effect was associated with increased autophagy, lysosome number, and exocytosis [25].

Oxidative stress is also an upstream stimulus that activates c-Abl and its target, the transcription factor p73, promoting apoptosis in NPC neurons [28,29,30]. Moreover, we demonstrated that supplementing the diet of NPC mice with the potent antioxidant α-Tocopherol (α-TOH, vitamin E) delays neurodegeneration. This treatment enhances Purkinje neurons survival, reduces astrogliosis and nitrotyrosine levels, and decreases the activation and phosphorylation of p73 in the cerebellum [31].

In this study, we explored the key TFEB Tyr(s) that are phosphorylated by c-Abl and mediate its cytoplasmic retention, and if the c-Abl/TFEB pathway is activated in other LSDs in addition to NPC. We analyzed Gaucher and NPA diseases, characterized by the primary accumulation of the lipids glucocerebroside and sphingomyelin, respectively, and secondly, by the accumulation of cholesterol. Moreover, considering the beneficial effects we had observed with the antioxidant treatment in NPC mice, we sought to evaluate the effect of α-TOH on TFEB localization and lysosomal clearance in these LSDs. We studied the pathological activation of c-Abl, the effect of α-TOH and two c-Abl inhibitors, Imatinib and Neurotinib, on TFEB localization and cholesterol accumulation. We also evaluated other potential Tyr candidates of TFEB to be phosphorylated by c-Abl and their relevance on the c-Abl/TFEB pathway.

Our results show that TFEB Tyr173 is key for c-Abl mediated cytoplasmic retention, and that activation of the c-Abl/TFEB pathway is a common pathogenic mechanism in neuronal models of Gaucher, NPA and NPC diseases. In addition, this study strongly supports that direct inhibition of c-Abl or preventing its activation through the use of an antioxidant agent, such as α-TOH, are therapeutic options for these three and other LSDs.

## 2. Materials and Methods

### 2.1. Cell Cultures and Treatments

The SH-SY5Y neuroblastoma cell line obtained from ATCC (Cat. CRL-2266, Manassas, VA, USA) was maintained in Dulbecco’s modified Eagle’s medium (DMEM) supplied with 10% Fetal bovine serum (FBS). The cells were pre-treated with Imatinib 10 μM or Neurotinib 1 μM or α-TOH (Merk Millipore Cat. T1157, Burlington, MA, USA) 0.1 mM by 1 h. For each experiment, we used a fresh α-TOH solution. Later, the cells were treated with conduritol-β-epoxide (CβE) (Merk Millipore Cat. 234599, Burlington, MA, USA) 150 μM for 5 days, Ds (Merk Millipore Cat. D3900, Burlington, MA, USA) 10 µM for 24 h, or with U18 (Enzo Life Science Inc. Cat. BML-S200, Farmingdale, NY, USA) 1 µg/mL for 24 h. The HeLa cell line was obtained from Dr. Ballabio (Telethon Institute of Genetics and Medicine (TIGEM), Italy) and was maintained in DMEM supplied with 10% FBS. The HeLa cells were treated with Torin-1 (Tocris Cat. 4247, Bristol, UK) 0.6 μM for 3 h, as a positive control for TFEB nuclear translocation.

### 2.2. Plasmids and Transfection

TFEB-GFP wild type (WT) was obtained from Andrea Ballabio’s laboratory (TIGEM, Italy). The different variants of TFEB-GFP were constructed in our laboratory (Table 1). The TFEB reference sequence accession number used in the plasmid construct is NM_007162.2. The plasmids containing TFEB-GFP and its respective mutants were transfected into HeLa cells using Lipofectamine 3000 transfection reagents (Thermo Fisher Scientific, Cat. L3000001, Waltham, MA, USA) for 48 h. At the end of the treatments, the cells were fixed and prepared for immunofluorescence, visualization using a Nikon Eclipse Ci-L (Tokyo, Japan) epifluorescence microscope, and analyses were run by the Fiji software (version 1.54f).

### 2.3. Immunofluorescence Staining

Cell lines were seeded in coverslips in 24-well culture plates. After treatment, the cells were washed with PBS and fixed in 4% paraformaldehyde/4% sucrose in PBS for 20 min at room temperature. Then, the cells were permeabilized in 0.2% Triton X-100 in PBS for 10 min. After two washes with PBS, the cells were incubated in 3% BSA in PBS for 30 min at room temperature. Then, the cells were incubated with anti p-c-Abl (Sigma, Cat. C5240, 1:850, St. Louis, MO, USA) or anti TFEB (Bethyl, Cat. 672A, 1:250, Montgomery, TX, USA) antibodies at 4 °C overnight. The cells were visualized using a Nikon Eclipse Ci-L (Tokyo, Japan) epifluorescence microscope or a Nikon Timelapse Ti-2E (Tokyo, Japan) confocal microscope and analyzed with the Fiji software.

### 2.4. Filipin Staining

The cells were fixed in 4% paraformaldehyde/4% sucrose in PBS for 20 min. Then, they were washed in PBS and treated with 1.5 mg/mL glycine for 20 min. After this, the cells were treated with 25 μg/mL Filipin (Merk Millipore Cat. F9765, Burlington, MA, USA) for 30 min and washed with PBS. The images were acquired with a Nikon Timelapse Ti-2E (Tokyo, Japan) confocal microscope and analyzed with the Fiji software.

### 2.5. Western Blotting

SH-SY5Y cells were lysed in RIPA buffer (25 mM Tris-HCl pH 7.6, 150 mM NaCl, 1% NP-40, 1% sodium deoxycholate, 0.1% SDS), supplemented with a cocktail of protease and phosphatase inhibitors (Thermo Fisher Scientific, Cat. 78442, Waltham, MA, USA). The homogenates were kept on ice for 10 min and then centrifuged at 10,000× *g* for 10 min (4 °C) to remove large debris. The supernatant was recovered, and protein concentration was determined with the Pierce BCA protein assay kit (Thermo Fisher Scientific, Cat. 23225, Waltham, MA, USA). The proteins were resolved by SDS-PAGE, transferred to a PVDF membrane, blocked, and incubated overnight at 4 °C with primary antibodies: rabbit anti-p-c-Abl (Sigma, Cat. C5240, 1:1000, St. Louis, MO, USA) and mouse anti-GAPDH (Santa Cruz, Cat. 32233, 1:3000, Dallas, TX, USA). The reactions were followed by incubation with peroxidase-conjugated secondary antibodies and developed using ECL technique (Thermo Fisher Scientific, Cat. 32106, Waltham, MA, USA).

### 2.6. Statistical Analysis

The number of independent experimental replicates, tests used, and the *p* values are indicated in each figure legend. Briefly, statistical analyses were performed using the Prism8 software (GraphPad Software Inc. Version 8.0.2). The data normality were checked using the Shapiro–Wilk test; all data showed normal distribution. One-way ANOVA test followed by Bonferroni post-test was used for comparison between more than two groups. *p* < 0.05 was considered statistically significant. In all graphs, the data represent the mean ± SEM.

## 3. Results

### 3.1. The Inhibition of c-Abl Induces TFEB Nuclear Localization in Gaucher, Niemann-Pick Type A, and Niemann-Pick Type C Neuronal Models

We have previously shown that c-Abl is activated in different models of Gaucher, NPA, and NPC diseases [26,28,29]. Here, we evaluated the c-Abl/TFEB pathway in neuronal models of Gaucher and NPA diseases, and included NPC models as positive control. To generate pharmacological neuronal models, we induced the Gaucher, NPA, and NPC phenotypes in human SH-SY5Y neuroblastoma cells. The SH-SY5Y cell line is extensively used as a neuronal model due to its ease of culture and expression of key neuronal markers relevant to neurodegenerative diseases such as PD and AD [32].

**Figure 1:**
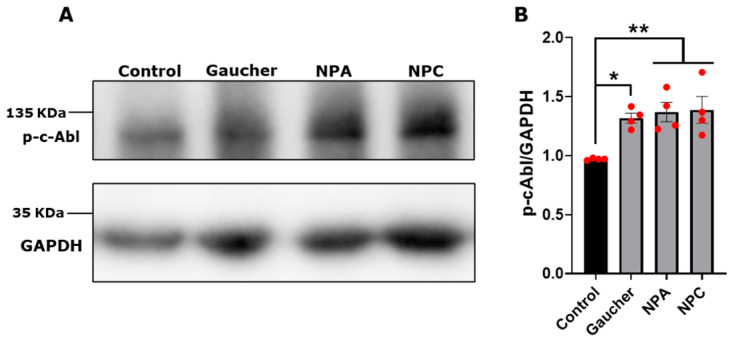
c-Abl activation in Gaucher, NPA, and NPC neuronal models. (**A**) The SH-SY5Y cells were treated with 150 μM CβE for 5 days, 10 μM Ds for 24 h, and 1 μg/mL U18 for 24 h for obtaining the Gaucher, NPA, and NPC models, respectively. Immunoblot analysis for p-c-Abl was performed. GAPDH was used as a loading control. (**B**) Quantification of p-c-Abl/GAPDH protein levels. A significant increase was observed in Gaucher, NPA, and NPC cells. The bars represent mean ± SEM (* *p* < 0.05; ** *p* < 0.01 One-way ANOVA Bonferroni Post hoc test, N = 4 independent experiments). The red dots indicate the biological replicates (independent experiments) for each condition.

We used the following treatments: (i) for Gaucher, 150 μM conduritol-β-epoxide (CβE), an irreversible competitive inhibitor of GCase, for 5 days [29]; (ii) for NPA, 10 μM desipramine (Ds), a functional inhibitor of acid sphingomyelinase (ASM) [33], for 24 h [26]; and (iii) for NPC, 1 μg/mL U18666A ((3β)-3-[2-(Diethylamino)ethoxy] androst-5-en-17-one hydrochloride) (U18), a cationic amphiphile molecule that prevents cholesterol egress from lysosomes by inhibiting Niemann-Pick C1 protein (NPC1) [34], for 24 h [25]. Next, we analyzed the levels of the activated phosphorylated form of c-Abl (p-c-Abl). We observed a significant increase in p-c-Abl levels in the Gaucher, NPA, and NPC neuronal models compared with the control cells (Figure 1A,B).

**Figure 2:**
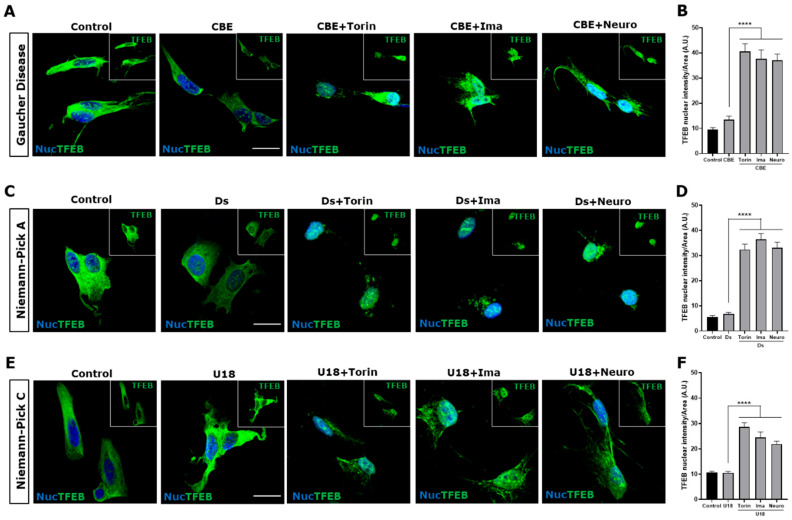
The inhibition c-Abl induces TFEB nuclear translocation in the Gaucher, NPA, and NPC neuronal models. Immunofluorescence staining of TFEB in Gaucher (**A**), NPA (**C**), and NPC (**E**) SH-SY5Y neuronal cell models obtained by treating with 150 μM CβE, 10 µM Ds, and 1 µg/mL U18, respectively. As a positive control for TFEB nuclear translocation, the cells were treated with Torin-1 (Torin) at 0.6 µM for 3 h before fixing the cells. Also, the cells were treated with 10 µM Imatinib (Ima) and 1 µM Neurotinib (Neuro). TFEB staining was visualized by Nikon Timelapse Ti-2E confocal microscope. For nuclear (Nuc) visualization, Hoechst staining was performed. The inset shows TFEB without Nuc staining. The scale bar is 20 μm. The graphs show the quantification of TFEB nuclear intensity for Gaucher (**B**), NPA (**D**), and NPC (**F**). The bars represent mean ± SEM (**** *p* < 0.0001, One-way ANOVA Bonferroni Post Hoc test, N = 3 independent experiments).

To study TFEB activation in the three disease models, we evaluated its nuclear localization under different conditions using immunofluorescence and confocal microscopy. Torin-1 0.6 μM for 3 h, a mTORC1 inhibitor, was used as a positive control for TFEB nuclear translocation (Figure 2). We observed that in control and Gaucher cells, TFEB was located mainly at the cytoplasm, meanwhile both c-Abl inhibitors, Imatinib and Neurotinib, as well as Torin-1, promoted a significant increase in TFEB nuclear levels in Gaucher conditions (Figure 2A,B). In the NPA neuronal model, we also observed a significant increase in the TFEB nuclear signal in the presence of c-Abl inhibitors and in the positive control cells treated with Torin-1 (Figure 2C,D). Confirming our previous results [25], in the NPC neuronal model, we observed that TFEB distribution was mainly cytoplasmatic, and an increase in its nuclear localization was observed after treatment with the c-Abl inhibitors Imatinib and Neurotinib (Figure 2E,F). Together, these results indicate that inhibiting c-Abl induces an increase in TFEB nuclear localization in these three neuronal models of LSDs.

### 3.2. TFEB Tyr173 Is Relevant for Its Cytoplasmic Retention Mediated by c-Abl

**Figure 3:**
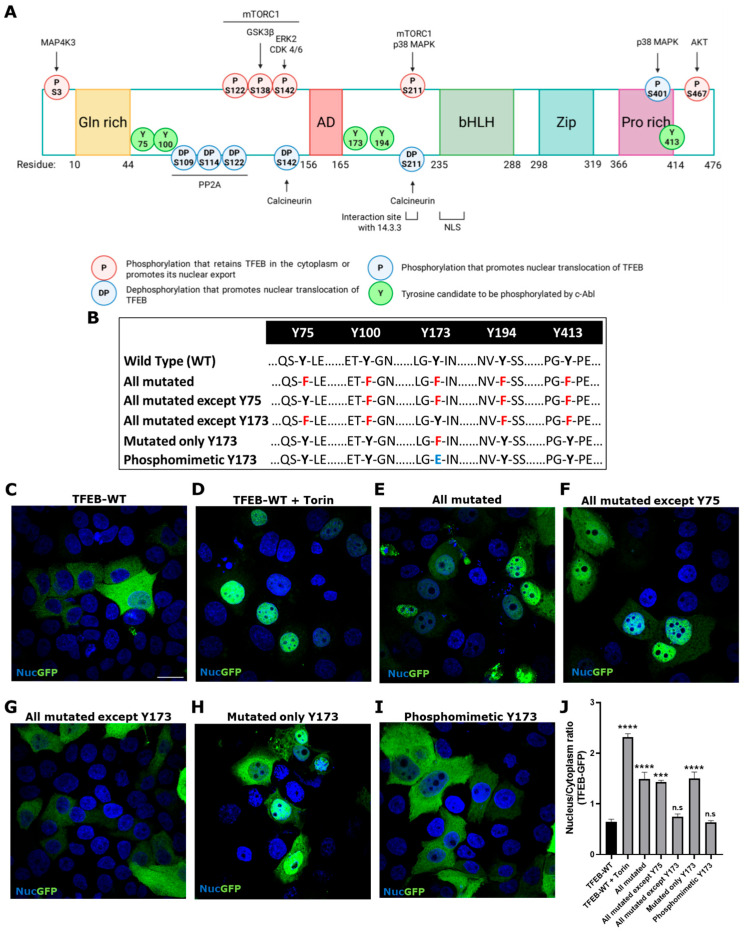
TFEB tyrosine phosphorylation at Y173 is key for its retention at the cytoplasm. (**A**) Schematic representation of regulation of TFEB subcellular localization by phosphorylation. The sites of TFEB regulation by phosphorylation are shown, with those that arrest TFEB in the cytoplasm or promote nuclear export highlighted in red, and those that promote nuclear translocation in blue. The kinases involved are indicated upper the phosphorylation site. The location of Y candidates for c-Abl phosphorylation are shown in green. The five TFEB domains are highlighted, the glutamine-rich domain [Gln-rich] in yellow, the transcriptional activation domain [AD] in red, the basic helix-loop-helix [bHLH] domain in green, the leucine zipper [Zip] domain in light blue, and the proline-rich [Pro-rich] domain in purple. The sites of interaction with 14.3.3 protein (residue 209–215) and the Nuclear Localization Signal (NLS, residue 230–250) are also indicated. Created with BioRender.com. (**B**) The Table shows TFEB-GFP wild type (WT) and the different variants; in red, mutation of Tyr [Y] to Phe [F]; in blue, mutation of Y to Glu [E]. HeLa cells were transfected with TFEB-GFP WT and the different variants for 48 h. TFEB-GFP and the variants were visualized using a Nikon Eclipse Ci-L epifluorescence microscope. Representative images of the subcellular localization of TFEB-GFP WT (**C**), TFEB-GFP WT treated with positive control for TFEB nuclear translocation Torin-1 (Torin) (**D**), all Tyr residues mutated (**E**), all Tyr residues mutated except Y75 (**F**), all Tyr residues mutated except Y173 (**G**), only Y173 mutated (**H**) and phosphomimetic Y173 mutated (**I**). Scale bar 10 μm. For nuclear (Nuc) visualization, Hoechst staining was performed. (**J**) The graph shows quantification of the nucleus/cytoplasm ratio fluorescence in different conditions. Values > 1 indicate TFEB nuclear translocation. The bars represent mean ± SEM (*** *p* < 0.001; **** *p* < 0.0001 One-way ANOVA Bonferroni Post hoc test, N = 3 independent experiments).

As previously mentioned, phosphorylation is the main TFEB modification that modulates its translocalization between the cytoplasm and nucleus [2,7]. The mTORC1 kinase complex phosphorylates TFEB at Ser 122, 138, 142, and 211, inducing its cytoplasmic retention [18,19,20]. Various Ser/Thr-Kinases, including among others, glycogen GSK3β, ERK1/2, and AKT, have been described as being capable of phosphorylating TFEB, resulting in its inactivation and cytoplasmic retention [21,22,23] (Figure 3A). Since c-Abl modulation regulates TFEB translocation, and our group has previously shown that c-Abl phosphorylates TFEB, we aimed to further investigate the relevance of phosphorylation at different Tyr residues. This was carried out through an in vitro phosphorylation assay using TFEB-Flag, recombinant human active c-Abl, and ATP-γ-^32^P [25]. In order to determine which Tyr could be the candidates to be phosphorylated by c-Abl, in silico studies were carried using the Netphos 2.0 platform, which allows us to search for consensus tyrosine phosphorylation sites [35], and the GPS 2.1.2 system to find possible specific phosphorylation sites for c-Abl [36]. Of the nine TFEB Tyr, we discarded those that do not have the consensus sequence for phosphorylation by c-Abl, leaving Tyr75, 100, 173, 194, and 413 as candidates (Figure 3A,B and Table 1). Previously, we showed that in cells transfected with TFEB variants that have Tyr (Y) 75 or Y173 changed to phenylalanine (Phe, F), Y75F, and Y173F, respectively, only the Y173F variant was preferentially found in the nucleus under physiological conditions compared to TFEB wild type (WT). Meanwhile, TFEB with the Y75F change increases slightly in the nucleus, also compared to TFEB WT [25], suggesting that Y173 and Y75 may be possible candidates to be phosphorylated by c-Abl. However, this evidence was not sufficient to rule out the implication of the other Tyr. Therefore, in this work, we aimed to determine which candidate Tyr residues are critical in the c-Abl/TFEB signaling pathway. To investigate this, we used TFEB variants fused with green fluorescent protein (GFP) to assess their phosphorylation by c-Abl and their impact on TFEB cytoplasmic retention (Figure 3A and Table 1). The variants included Phosphomimetic (Y173E-TFEB-GFP), which mimics phosphorylation by c-Abl, as the glutamic acid (Glu, E) residue carries a negative charge at physiological pH, similar to phosphorylated Tyr. All Tyr residues mutated (Y75F-Y100F-Y173F-Y194F-Y413F-TFEB-GFP): this variant prevents phosphorylation at these five amino acids. All Tyr residues mutated, except Y75 (Y100F-Y173F-Y194F-Y413F-TFEB-GFP): this variant allows only Y75 to be phosphorylated. All Tyr mutated, except Y173 (Y75F-Y100F-Y194F-Y413F-TFEB-GFP): this variant allows only Y173 to be phosphorylated. Only Y173 mutated (Y173F-TFEB-GFP): this variant prevents phosphorylation at Y173 while leaving the other Tyr residues susceptible to phosphorylation.

To determinate the subcellular localization of TFEB, HeLa cells were employed due to their high transfection efficiency and robust growth characteristics. Their well-established use in molecular and cellular biology makes them an ideal system for evaluating subcellular localization of proteins under controlled experimental conditions [37].

The HeLa cells were transfected with the different variants described in Figure 3A,B and Table 1. As expected, WT TFEB-GFP is predominantly localized in the cytoplasm (Figure 3C). Torin-1 was used as a positive control to induce TFEB nuclear localization; therefore, the WT variant significantly increases its nuclear localization (Figure 3D,J). Likewise, the variants carrying all the Tyr residues mutated and all mutated except Y75 show significant nuclear localization (Figure 3E,F,J). On the other hand, the variant with all Tyr mutated, except Y173, presents significant cytoplasmic localization (Figure 3G,J). Interestingly, we observed that the TFEB variant carrying only Y173 mutated has a constitutive nuclear localization compared to the WT (Figure 3H,J). The phosphomimetic variants significantly increase in the cytoplasm (Figure 3I,J).

These results strongly support that TFEB phosphorylation at Y173 is key for its retention in the cytoplasm.

### 3.3. The Inhibition of c-Abl Induces Cellular Clearance of Cholesterol in Gaucher, Niemann-Pick Type A, and Niemann-Pick Type C Neuronal Models

We have previously shown that, in NPC models, the inhibition of c-Abl increases TFEB nuclear localization. This effect is associated with increased expression of TFEB target genes, autophagic flux, and lysosome exocytosis, which promotes cholesterol clearance [25].

Gaucher, NPA, and NPC cells accumulate primary glucocerebroside, sphingomyelin, and cholesterol, respectively. Gaucher and NPA also accumulate other secondary lipids, such as cholesterol [38,39]. Therefore, we decided to study the effect of c-Abl inhibition on cholesterol accumulation in Gaucher and neuronal models using NPC as a positive control.

**Figure 4:**
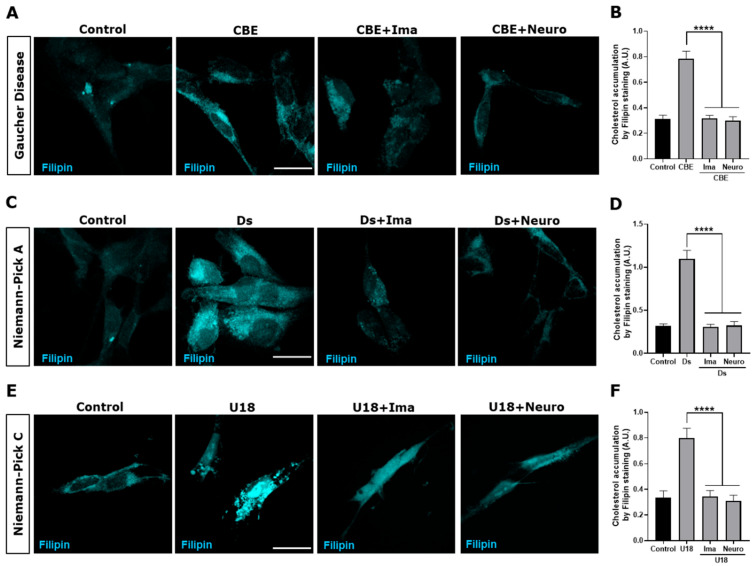
c-Abl inhibition decreases cholesterol accumulation in the Gaucher, NPA, and NPC neuronal models. The SH-SY5Y cells were treated with 150 μM CβE for 5 days, 10 μM Ds for 24 h, and 1 μg/mL U18 for 24 h to develop the Gaucher (**A**), NPA (**C**), and NPC (**E**) models, respectively. Additionally, the cells were pre-treated with Imatinib (Ima) 10 µM or Neurotinib (Neuro) 1 μM for 1 h and maintained for 24 h until the end of the experiment. Next, 30 min after fixing the cells, they were stained with filipin and visualized using a Nikon Timelapse Ti-2E confocal microscope. The scale bar was 10 μm. The graph shows the quantification of cholesterol accumulation by filipin staining in Gaucher (**B**), NPA (**D**), and NPC (**F**) models treated with the c-Abl inhibitors Ima or Neuro. The bars represent mean ± SEM (**** *p* < 0.0001 One-way ANOVA Bonferroni Post hoc test, N = 3 independent experiments).

We followed cholesterol accumulation using filipin staining. As expected, an increase and a more clustered perinuclear distribution of the filipin signal were observed in cells treated with CβE (Figure 4A), Ds (Figure 4C), and U18 (Figure 4E) compared with the control untreated cells. Interestingly, we observed a significant decrease in filipin staining when the Gaucher (Figure 4B), NPA (Figure 4D), and NPC (Figure 4F) cells were treated with the c-Abl inhibitors Imatinib and Neurotinib. These results support the idea that c-Abl inhibition induces cellular clearance in LSDs that accumulate lipids. Taken together, these results show that the activation of the c-Abl/TFEB pathway is a common pathogenic mechanism in LSDs and that c-Abl inhibition promotes cellular clearance of stored substrates.

### 3.4. α-TOH Inhibits c-Abl Activation and Induces TFEB Nuclear Translocation in an NPC HeLa Model

**Figure 5:**
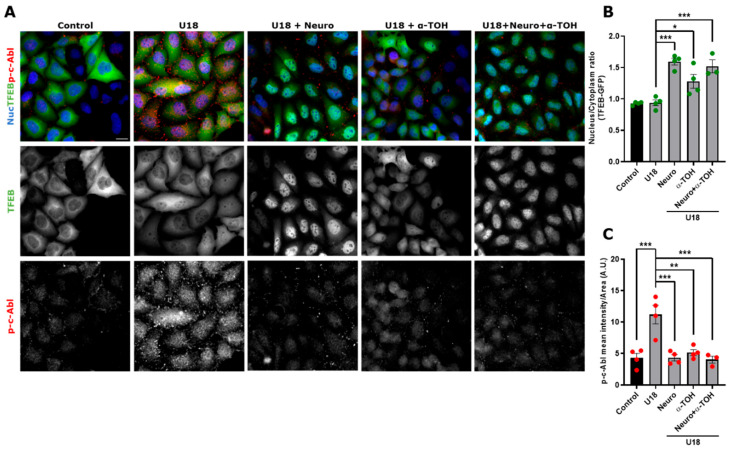
α-TOH inhibits c-Abl activation and induces TFEB nuclear translocation in an NPC HeLa cell model. (**A**) Representative images of HeLa cells transfected with TFEB-GFP for 48 h and then treated with 1 μg/mL U18 for 24 h for obtaining the NPC model. The cells were pre-treated with Neurotinib (Neuro) 1 μM and/or α-TOH 0.1 mM for 1 h and maintained for 24 h until the end of the experiment. Next, the samples were stained for p-c-Abl. TFEB-GFP and p-c-Abl were visualized using a Nikon Eclipse Ci-L epifluorescence microscope. For nuclear (Nuc) visualization, Hoechst staining was performed. The scale bar was 20 μm. (**B**) Quantification of the nucleus/cytoplasm ratio under different conditions. Values > 1 indicate TFEB nuclear translocation. The bars represent mean ± SEM (* *p* < 0.05; *** *p* < 0.001, One-way ANOVA Bonferroni Post Hoc test, N = 4 independent experiments). (**C**) Quantification of p-c-Abl intensity in the presence of Neuro or α-TOH; or Neuro and α-TOH. The bars represent mean ± SEM (** *p* < 0.01; *** *p* < 0.001, One-way ANOVA Bonferroni Post Hoc test, N = 4 independent experiments). The red and green dots indicate the biological replicates (independent experiments) for each condition. Green for TFEB and red for p-c-Abl.

Our labs and other researchers have shown that oxidative stress activates proapoptotic c-Abl functions in neuronal models [28,40]. To analyze if α-TOH can inhibit the c-Abl/TFEB pathway and induce TFEB nuclear localization, we transfected HeLa cells with the TFEB-GFP plasmid for 48 h, after which the cells were treated with U18 to induce the NPC pharmacological model, α-TOH, and Neurotinib (Figure 5A). Interestingly, we observed that treating NPC cells with α-TOH induces TFEB translocation to the nucleus, similar to the response we found when c-Abl was inhibited with Neurotinib. We also observed that combining Neurotinib with α-TOH does not produce a synergistic effect on TFEB nuclear localization (Figure 5A,B).

Next, we assessed whether activation of c-Abl is affected by α-TOH in the NPC model. In TFEB-GFP-HeLa cells treated with U18, we observed that, in the presence of α-TOH, the p-c-Abl signal decreased to similar levels as control and Neurotinib conditions (Figure 5A,C). We did not observe a synergistic effect when the cells were treated with Neurotinib and α-TOH in the NPC context (Figure 5C). Our results suggest that α-TOH reduces c-Abl activation and promotes TFEB nuclear localization in the HeLa cell NPC model.

## 4. Discussion

Here, we show that c-Abl and the c-Abl/TFEB pathway are activated in pharmacological neuronal models of Gaucher, NPA, and NPC diseases. Also, we showed that Tyr173 is required for cytoplasmic retention of TFEB, indicating that its phosphorylation could be the mechanisms by which c-Abl activity contributes to negatively modulate TFEB function. Therefore, c-Abl inhibition, by Imatinib and Neurotinib, induces increased TFEB levels in the nucleus and decreased cholesterol accumulation in the three LSDs neuronal models. Interestingly, in NPC cells, the decrease in p-c-Abl signal intensity induced by α-TOH correlates with TFEB nuclear translocation

Imatinib is a classic c-Abl inhibitor that binds to its ATP binding domain [41] and has poor distribution towards the brain [42]. On the other hand, Neurotinib, a new selective c-Abl inhibitor developed by our group, binds at the myristate allosteric pocket, has appropriate pharmacokinetics and high central nervous system permeability [26,43]. The strong decrease in pathological c-Abl activation, accompanied by increased TFEB nuclear translocation and reduced cholesterol accumulation, supports the role of the c-Abl/TFEB pathway in lysosomal clearance in pharmacological models of LSDs. Interestingly, Neurotinib, with its better brain penetrance, improves cognitive function and decreases neuronal loss in hippocampus and cortex in a NPA in vivo mouse model, supporting the potential use of this inhibitor in patients suffering from NPA, other LSDs, or neurodegenerative diseases [26]. In this study, we utilized the human neuroblastoma cell line SH-SY5Y to establish pharmacological models of Gaucher, NPA, and NPC diseases, enabling the evaluation of c-Abl/TFEB pathway activation and its impact on lysosomal clearance. SH-SY5Y is widely utilized as an in vitro neuronal model for studying neurodegenerative diseases due to its ease of culture, high proliferative capacity, and because this cellular line exhibits various neuronal markers [44]. Given these characteristics, SH-SY5Y cells offer a practical system for investigating lysosomal dysfunction and neurodegenerative mechanisms. However, their tumorigenic origin confers properties such as uncontrolled proliferation and incomplete maturation limit their ability to replicate the functional properties of mature human neurons [45] and should therefore be considered when analyzing the results obtained. For the Gaucher cellular model, we used CβE, an irreversible competitive inhibitor of GCase, the lysosomal enzyme whose deficiency leads to Gaucher disease. It is widely used in research to generate cellular and animal models that closely mimic the disease, facilitating the study of potential therapeutic strategies [46]. However, it has some specific limitations. Unlike genetic models, such as the knockout (KO) mice for the GBA gene encoding for GCase, CβE treatment does not fully eliminate GCase enzymatic activity, potentially resulting in a residual function that does not accurately reflect the severe deficiency observed in patients [47]. However, mouse models of the neuronopathic type of Gaucher disease, showing neurological signs, have a short lifespan, usually dying within one month after [48], making difficult to conduct studies on them. Interestingly, a mouse model of neuronopathic Gaucher was recently described, with a significantly extended lifespan and progressive neurological impairment [49], opening up options to study this form of the disease in this model as well as to explore therapeutic options. In addition, patient-derived induced pluripotent stem cells (iPSCs) provide a more faithful model, as they retain the patients’ genetic background, including specific *GBA1* mutations and modifier variants, allowing differentiation into disease-relevant cell types such as macrophages and neurons [50]. Therefore, although CβE-based models are valuable for mechanistic studies and screening, complementary use of genetic and iPSC-derived models remains essential for a comprehensive understanding of Gaucher disease pathophysiology and is necessary to project the findings of this work.

Oxidative stress is a major factor involved in the physiopathology of LSDs, particularly in neurodegeneration [51,52]. Lysosomal dysfunction, for example, by lipid accumulation, downregulates mitophagy, preventing adequate mitochondrial homeostasis, promoting oxidative stress [53]. Oxidative stress plays a role in NPC cellular and mouse models, and the use of antioxidants can delay neurodegeneration in NPC mice [28,31]. Importantly, we have previously shown that in the NPC mouse model, oxidative stress induces c-Abl activation, while antioxidants such as N-Acetylcysteine or α-TOH improve the phenotype by reducing c-Abl/p73 signaling, enhancing neuronal survival and alleviating neurological symptoms [31]. α-TOH, a member of the Vitamin E phenolic group, is the primary natural lipid antioxidant that protects fatty acids from reactive oxygen species (ROS), reactive nitrogen species (RNS), and lipid peroxidation [54,55]. This antioxidant is widely used as a nutritional supplement for various conditions due to its potential beneficials effects [55,56,57]. As we have shown, α-TOH reduces pathological c-Abl activation and increases TFEB nuclear localization in an NPC model, similar to c-Abl inhibitors. Nevertheless, we cannot exclude the possibility that α-TOH is involved in other signaling pathways contributing to these effects [58]. As a relevant transcription factor in the antioxidant mechanism of the cell, TFEB regulates a positive circuit of genes that promotes the ROS reduction [59]. Additionally, oxidation at cysteine 212 (C212) has been shown to promote TFEB nuclear localization [60]. Interestingly, the reduction in oxidative stress by α-TOH, promotes the nuclear localization of TFEB, possibly through a positive feedback mechanism.

Although the use of α-TOH is beneficial, the effects of the c-Abl inhibitor Neurotinib exceed those of this nutraceutical in the inactivation of p-c-Abl and nuclear localization of TFEB in NPC models. Further studies are needed to analyze the impact of α-TOH and other antioxidants on the accumulation of cholesterol in these LSDs.

As we have shown, the use of c-Abl inhibitors reduces the accumulation of cholesterol characteristic of these LSDs. These results can be interpreted as an increase in cellular clearance, opening the possibility of using c-Abl inhibitors to treat LSDs that accumulate non-degraded substrates in the lysosome. In line with this idea, it has been shown that in Pompe LSD, where there is accumulation of glycogen, overexpression of TFEB improves cellular clearance [22,61]. Given that c-Abl is active in AD, PD, Amyotrophic Lateral Sclerosis (ALS), and Frontotemporal Dementia (FTD) diseases [62,63,64], and that it has also been shown that TFEB overexpression and/or activation is beneficial [22,61,65,66], inhibiting c-Abl in order to increase TFEB in the nucleus could be a therapeutic option for these neurodegenerative and more frequent diseases. Moreover, the therapeutic relevance of targeting the c-Abl/TFEB pathway in vivo has been previously demonstrated in the NPC mouse model, where treatment with c-Abl inhibitors Imatinib or GNF2 enhanced TFEB nuclear translocation and reduced cholesterol accumulation in brain tissue [25].

TFEB, as the master regulator of the CLEAR network, is involved in lysosomal biogenesis, exocytosis, and autophagy [8]. In LSDs, lysosome dysfunction results in accumulation of these organelles caused by the buildup of unmetabolized substrates in them, generating cellular stress. Increasing the activity of TFEB leads to an improvement in these pathways, although the mechanism of TFEB-activated pathways remains to be investigated. We speculate that it is highly probable that the decrease in accumulated cholesterol and other metabolites in these LSDs is driven by their exocytosis. Further studies are needed to explore this hypothesis.

To determine which Tyr could be the candidate to be phosphorylated by c-Abl and to further investigate the role of this Tyr in the c-Abl/TFEB signaling pathway, we created various TFEB-GFP variants. Using Netphos 2.0 and GPS 2.1.2 platforms, five Tyr candidates (Tyr75, 100, 173, 194, and 413) were selected based on their potential for being phosphorylation targets of c-Abl. We had previously explored the relevance of two of these five Tyr candidates, specifically Y75 and Y173, and found that mutation at Y75 and Y173 influenced TFEB localization, with the Y173F variant preferentially found in the nucleus compared to Y75F, which was found mostly in the cytoplasm under physiological conditions [25]. In the present study, HeLa cells were transfected with different variants of TFEB fused to GFP to assess their subcellular localization. Interestingly, both the fully mutated variant (all candidate Tyr mutated) and the variant in which the only non-mutated Tyr residue was Y75 showed significant nuclear localization, indicating that phosphorylation of Tyr75 is not crucial for TFEB retention in the cytoplasm. In contrast, the variant excluding Y173 (all mutated except Y173) exhibited a strong cytoplasmic localization, whereas the Y173F mutation (only Y173 mutated) resulted in a constitutive nuclear presence, reinforcing the significance of Y173 phosphorylation. Overall, these findings strongly suggest that TFEB Tyr173 is phosphorylated by c-Abl and is essential for its retention in the cytoplasm. Our results highlight Tyr173’s crucial role in regulating TFEB localization, contributing to a deeper understanding of TFEB’s regulatory mechanism and its interaction with c-Abl. Further studies are necessary to determine the relevance of Tyr173 of TFEB under pathogenic conditions, such as the LSDs studied in this work.

Our results, using experiments with mutants, indirectly demonstrate that Tyr173 of TFEB is the key c-Abl phosphorylation site. Direct evidence, such as mass spectrometry and in vitro kinase assays or using phospho-specific antibodies against Tyr173 are required to sustain that this is the key Tyr of TFEB phosphorylated by c-Abl that controls its subcellular localization. These approaches are challenging because of the lability of Tyr phosphorylation for mass spectrometric analysis and the need for an antibody specific for phosphorylated Tyr173. Given that TFEB nuclear translocation is commonly regulated by mTORC1-dependent Ser phosphorylation, it was important to assess whether our observation could result from alterations at these canonical sites. Previous studies of our group demonstrate that c-Abl regulates TFEB nuclear translocation independently of mTORC1-mediated Ser phosphorylation. In addition, we showed that c-Abl inhibition with Imatinib promotes a partial and different TFEB de-phosphorylation status compared with the inhibition of mTORC1, supporting a distinct regulatory mechanism [25]. Altogether, these results suggest a direct regulatory role of c-Abl in controlling TFEB subcellular localization. However, it is also plausible that lysosomal stress or lipid accumulation, common features of LSDs, may act upstream to activate c-Abl, which in turn modifies TFEB activity. Indeed, lysosomal dysfunction is known to generate oxidative stress, a well-established activator of c-Abl in neuronal contexts [27]. There are no antecedents of an interaction between c-Abl and damaged lysosomes, directly or through other proteins. Interestingly, we have recently shown that c-Abl is activated and localized in mitochondria in response to endoplasmic reticulum (ER) stress and collaborates in mitochondrial fragmentation [67], supporting that c-Abl subcellular localization can change in response to particular cellular stress or signals. Further studies are needed to analyze the relationship between lysosomal damage, c-Abl activation, and TFEB phosphorylation.

Finally, our results show that lysosomal clearance can be induced through the activation of TFEB by inhibiting the tyrosine kinase c-Abl in cellular models of Gaucher, NPA, and NPC diseases. Our data support the possibility of treating LSD patients and patients suffering from other neurodegenerative diseases in which c-Abl is activated and lysosomal function is compromised with c-Abl inhibitors.

## 5. Conclusions

In summary, our findings demonstrate that the activation of the c-Abl/TFEB pathway represents a common pathogenic mechanism in pharmacological cellular models of Gaucher, NPA, and NPC diseases. We identified TFEB Tyr173 as a key residue for c-Abl-mediated phosphorylation, regulating its subcellular localization and function. Pharmacological inhibition of c-Abl using Imatinib, the brain-penetrant inhibitor Neurotinib, and antioxidant intervention with α-Tocopherol promote TFEB nuclear localization, enhance lysosomal clearance, and reduce pathological lipid accumulation. These results highlight the therapeutic potential of targeting the c-Abl/TFEB pathway to restore lysosomal function in lysosomal storage disorders and suggest broader implications for neurodegenerative diseases associated with oxidative stress and lysosomal dysfunction.

## Figures and Tables

**Table 1:** Sequences used in this study for mutation in TFEB plasmid (human).

TFEB	Sequence	Y75	Y100	Y173	Y194	Y413
**Wild Type (WT)**	Aa	QS**Y**LE	ET**Y**GN	LG**Y**IN	NV**Y**SS	PG**Y**PE
	Codon	CAGTCC**TAC**CTGGAG	GAGACC**TAT**GGGAAC	CTTGGC**TAC**ATCAAT	AATGTG**TAC**AGCAGC	CCGGGC**TAC**CCCGAA
**Phosphomimetic Y173**	Aa	QS**Y**LE	ET**Y**GN	LG**E**IN	NV**Y**SS	PG**Y**PE
	Codon	CAGTCC**TAC**CTGGAG	GAGACC**TAT**GGGAAC	CTTGGC**GAG**ATCAAT	AATGTG**TAC**AGCAGC	CCGGGC**TAC**CCCGAA
**All mutated** **except Y173**	Aa	QS**F**LE	ET**F**GN	LG**Y**IN	NV**F**SS	PG**F**PE
	Codon	CAGTCC**TTC**CTGGAG	GAGACC**TTC**GGGAAC	CTTGGC**TAC**ATCAAT	AATGTG**TTC**AGCAGC	CCGGGC**TTC**CCCGAA
**All mutated**	Aa	QS**F**LE	ET**F**GN	LG**F**IN	NV**F**SS	PG**F**PE
	Codon	CAGTCC**TTC**CTGGAG	GAGACC**TTC**GGGAAC	CTTGGC**TTC**ATCAAT	AATGTG**TTC**AGCAGC	CCGGGC**TTC**CCCGAA
**All mutated** **except Y75**	Aa	QS**Y**LE	ET**F**GN	LG**F**IN	NV**F**SS	PG**F**PE
	Codon	CAGTCC**TAC**CTGGAG	GAGACC**TTC**GGGAAC	CTTGGC**TTC**ATCAAT	AATGTG**TTC**AGCAGC	CCGGGC**TTC**CCCGAA
**Mutated only Y173**	Aa	QS**Y**LE	ET**Y**GN	LG**F**IN	NV**Y**SS	PG**Y**PE
	Codon	CAGTCC**TAC**CTGGAG	GAGACC**TAT**GGGAAC	CTTGGC**TTC**ATCAAT	AATGTG**TAC**AGCAGC	CCGGGC**TAC**CCCGAA

In red, mutation of Tyr [Y] to Phe [F]; in blue, mutation of Y to Glu [E]. Aa: amino acid.

## Data Availability

The data are contained within this article.
